# Correlations between arm motor behavior and brain function following bilateral arm training after stroke: a systematic review

**DOI:** 10.1002/brb3.411

**Published:** 2015-11-26

**Authors:** Pei Ling Choo, Helen L. Gallagher, Jacqui Morris, Valerie M. Pomeroy, Frederike van Wijck

**Affiliations:** ^1^ School of Health and Life SciencesGlasgow Caledonian UniversityGlasgowUK; ^2^ Nursing, Midwifery and Allied Health Professions Research UnitGlasgow Caledonian UniversityGlasgowUK; ^3^ Acquired Brain Injury Rehabilitation Alliance (ABIRA)NorwichUK; ^4^ University of East AngliaNorwichUK

**Keywords:** Bilateral, imaging, rehabilitation, review, Stroke, upper limb

## Abstract

**Background:**

Bilateral training (BT) of the upper limb (UL) might enhance recovery of arm function after stroke. To better understand the therapeutic potential of BT, this study aimed to determine the correlation between arm motor behavior and brain structure/function as a result of bilateral arm training poststroke.

**Methods:**

A systematic review of quantitative studies of BT evaluating both UL motor behavior and neuroplasticity was conducted. Eleven electronic databases were searched. Two reviewers independently selected studies, extracted data and assessed methodological quality, using the Effective Public Health Practice Project (EPHPP) tool.

**Results:**

Eight studies comprising 164 participants met the inclusion criteria. Only two studies rated “strong” on the EPHPP tool. Considerable heterogeneity of participants, BT modes, comparator interventions and measures contraindicated pooled outcome analysis. Modes of BT included: in‐phase and anti‐phase; functional movements involving objects; and movements only. Movements were mechanically coupled, free, auditory‐cued, or self‐paced. The Fugl‐Meyer Assessment (UL section) was used in six of eight studies, however, different subsections were used by different studies. Neural correlates were measured using fMRI and TMS in three and five studies, respectively, using a wide variety of variables. Associations between changes in UL function and neural plasticity were inconsistent and only two studies reported a statistical correlation following BT.

**Conclusions:**

No clear pattern of association between UL motor and neural response to BT was apparent from this review, indicating that the neural correlates of motor behavior response to BT after stroke remain unknown. To understand the full therapeutic potential of BT and its different modes, further investigation is required.

## Introduction

Stroke is the second highest cause of death and the leading cause of disability globally (Di Carlo 2009). Of those who survive, only a third regain some functional use of the upper limb (UL) (Kwakkel et al. 2003), which impacts on independence, mood and participation (Levy et al. 2001; Langhorne et al. 2009; Morris et al. 2013). Considering that most activities of daily living (ADL) involve the UL, it is crucial to improve UL motor behavior after stroke.

UL motor rehabilitation focuses mostly on unilateral training (i.e., training the affected UL only) (Winstein et al. 2004). Interventions include: electromyography biofeedback (Woodford and Price 2007), electrostimulation (Pomeroy et al. 2009), robotic‐assisted training (Mehrholz et al. 2012) and constraint‐induced therapy (CIT) (Sirtori et al. 2009). In contrast, bilateral UL training (BT) is a form of training where both ULs perform identical movements simultaneously, yet independently (e.g., carrying a box) (Mudie and Matyas 1996; Stewart et al. 2006). BT can be undertaken in different modes. In the in‐phase mode, both ULs move in the same direction at the same time (e.g., bending both elbows). In the antiphase mode, one UL moves in one direction (e.g., bending the elbow) as the other moves in the opposite direction (e.g., extending the elbow). BT is not to be confused with bimanual training, where both limbs move simultaneously but perform different movement patterns (e.g., tying shoelaces, opening a jar).

BT emerged from motor control theories and observations in nonimpaired people that, during rhythmic movement of both limbs, a coupling effect occurs in which both limbs adopt similar spatial and temporal movement characteristics, leading to a stable form of coordination (Kelso 1984; Swinnen 2002). Beneficial effects of BT are thought to arise from this interlimb coupling effect, which in people with stroke may lead to facilitation of paretic arm movement by the nonparetic arm (Mudie and Matyas 1996; Swinnen 2002; Stewart et al. 2006). It is postulated that the simultaneous activation of both hemispheres facilitates activation of the damaged hemisphere (Cauraugh et al. 2008; Stinear et al. 2008) through rebalancing of interhemispheric inhibition that has been disrupted following a stroke. BT is thought to reduce transcallosal inhibition from the nonaffected hemisphere to the affected hemisphere, thereby increasing output of the latter (Stinear and Byblow 2004; Cauraugh and Summers 2005). Additional pathways that may be facilitated during BT include ipsilateral uncrossed corticospinal pathways (Cauraugh and Summers 2005) and spared indirect corticospinal pathways, which receive input from bilateral reticulospinal and rubrospinal pathways (Mazevet 2003).

Individual studies with stroke survivors have reported benefits of BT including: increased velocity and smoothness of movement (Cunningham et al. 2002; Harris‐Love et al. 2005), long‐term functional recovery (Whitall et al. 2000; Luft et al. 2004; Cauraugh et al. 2008; Stinear et al. 2008) and changes in brain activation. BT has been recommended in the UK National Clinical Guidelines for Stroke for patients with persistent UL impairment (Intercollegiate Stroke Working Party, 2012). However, there is as yet no conclusive evidence from systematic reviews to show significant benefits of BT over unilateral UL training, placebo or no training (Stewart et al. 2006; Langhorne et al. 2009; Cauraugh et al. 2010; Coupar et al. 2010; Latimer et al. 2010; Van Delden et al. 2012; Pollock et al. 2014a). A Cochrane overview of systematic reviews found unilateral UL training to be more beneficial than bilateral UL training in terms of UL motor behavior and ADL, but no more beneficial in terms of arm motor behavior. However, the evidence was of moderate GRADE quality only (Pollock et al. 2014a).

The lack of conclusive evidence of the efficacy of BT has been attributed to the inadequate matching of diverse BT methods to patient characteristics and/or the use of inappropriate outcome measures (Sleimen‐Malkoun et al. 2011). It is conceivable that BT may have a restorative effect in some stroke survivors. Assessing the potential of BT as an intervention in stroke rehabilitation, therefore, requires a better understanding of the neural correlates of motor behavior response to BT. Moreover, given that stroke recovery is associated with reorganization of neural networks (Cramer and Bastings 2000; Calautti and Baron 2003), it is important to understand how BT impacts on neuroplasticity in order to advance knowledge of how to match rehabilitation interventions to individual patients. Over the last decade, several reviews have provided evidence for associations between arm motor recovery and neuroplastic changes in brain structure/function, using methods including transcranial magnetic stimulation (TMS) or Functional Magnetic Resonance Imaging (Calautti and Baron 2003; Ward 2006; Buma et al. 2010).

While there are other reviews of BT (Stewart et al. 2006; Langhorne et al. 2009; Cauraugh et al. 2010; Coupar et al. 2010; Latimer et al. 2010; Van Delden et al. 2012; Veerbeek et al. 2014), they have not addressed changes in neuroplasticity alongside changes in UL motor behavior. The aim of the systematic review reported here was to identify the relationship between arm motor behavior and brain structure/function in response to BT after stroke. To the authors' knowledge, this is the first systematic review to address the question of how BT affects UL motor behavior and neural function after stroke.

## Methods

### Design

A systematic review with pre‐set inclusion criteria, independent identification of studies and data extraction, and narrative synthesis.

### Inclusion criteria

#### Type of study

Quantitative studies of any design were included as this is a novel topic (Armstrong et al. 2007) and as studies reporting on neuroplasticity may include case series or small cohort studies.

#### Type of participants

Adults (≥18 years old) with a clinical diagnosis of stroke (WHO Monica Project Principal Investigators, 1988). Studies with participants at any time since stroke, with any type and locality of stroke, initial UL impairment, previous stroke(s), and comorbidities were included.

#### Type of intervention

Studies including any mode of BT were eligible, for example, in‐phase and anti‐phase, movements with and without objects. In the absence of a minimum standard for the dose of BT, we accepted studies that included at least a single session of any intensity, similar to the Cochrane systematic review on BT (Coupar et al. 2010). Only studies allowing the effects of BT to be analyzed as a single intervention were included.

#### Type of outcomes

Studies had to include both a measure of the effects of BT on: (1) UL motor behavior (e.g., Action Research Arm Test [ARAT], Fugl‐Meyer Assessment [FM]), and a measure of (2) neuroplasticity (change in brain structure or function, e.g., number of active voxels during a movement task, excitability of the corticospinal pathway).

### Exclusion criteria

Studies not available in English or in full text were excluded.

### Search strategy

Complete holdings of 11 databases were searched up until December 2014: Cochrane Stroke Group Trials Register, MEDLINE, EMBASE, CINAHL, AMED, PsycINFO, ProQuest Central, Web of Science, Physiotherapy Evidence database PEDro (http://www.pedro.org.au), OTseeker (http://www.otseeker.com/) and REHABDATA (http://www.naric.com/research/rehab/default.cfm).

To identify further published, unpublished and ongoing studies, ClinicalTrials.gov (http://www.clinicaltrials.gov/), Current Controlled Trials (www.controlled-trials.com), Stroke Trials Directory (www.strokecenter.org/trials/), Science Citation Index Reference Search, Index to Theses and ProQuest Dissertations and Theses were searched up until December 2014. Reference lists of included studies and relevant systematic reviews were also checked.

### Search terms

A combination of controlled vocabulary (MeSH) and free‐text terms relating to the condition “Stroke,” intervention “Simultaneous bilateral upper limb training” and body part “Upper limb” were used in the search strategy. These key words were modified to suit each database (see Appendix S1).

### Study identification

One review author (PLC) conducted the literature search and eliminated obviously irrelevant titles and duplicates. Two authors (PLC and FvW) independently read the abstracts of the remaining studies and applied the above inclusion and exclusion criteria to classify each as “definitely relevant,” “possibly relevant” or “definitely irrelevant.” Those labeled “possibly relevant” were classified through discussion. The authors then independently reviewed the full texts of the “definitely relevant” and “possibly relevant” studies and used the same criteria to classify each as “include,” “unsure,” or “exclude.” Studies which both reviewers classified as “include” and “exclude” were included and excluded in the review, respectively. The remaining studies were classified through discussion. Where a decision could not be agreed between the authors, the opinion of a third author (HG) was sought and consensus reached through discussion.

### Data analysis

#### Assessment of methodological quality

Two authors (PLC and FvW) independently assessed the methodological quality of the included studies using the Effective Public Health Practice Project (EPHPP) tool (Thomas et al. 2004). This tool was selected after considering the research questions and recommendations from four reviews (US Department of Health and Human Services, 2002; Deeks et al. 2003; Sanderson et al. 2007; Crowe and Sheppard 2011). Where necessary, a third author (HG) was involved to resolve any disagreement between PLC and FvW through discussion.

#### Data extraction

Data extraction pertaining to transcranial magnetic stimulation (TMS) was independently performed by two authors (PLC and VP). The same applied to fMRI data (PLC and HG). The remaining data were independently extracted by two authors (PLC and FvW). Reports of adverse events were also documented. All data extraction was conducted using standardized forms. If there were queries about the published reports then authors were contacted for clarification.

#### Data synthesis

Included studies were clustered according to whether they utilized either a single phase mode or a combination of in‐phase and anti‐phase modes of BT. If data were suitable for pooling, all outcome measures were to be analyzed as continuous data. Standardized mean differences (SMD) and 95% confidence intervals (CI) were also to be calculated. Heterogeneity was planned to be determined using the *I* ^2^ statistic. Meta‐analysis was planned as both fixed‐effect and random‐effects modeling to assess sensitivity to the choice of modeling approach. We also intended to undertake subgroup analyses for time since stroke, type of stroke, severity of arm impairment, and mode of BT. However, considerable heterogeneity of participants, BT modes, comparator interventions and measures emerged from the findings, which contraindicated the pooled outcome analyses, as well as the planned subgroup analyses. A narrative synthesis was therefore performed. Publication bias was assessed where there were more than ten studies included for review.

## Results

### Characteristics of included studies

**Figure 1:**
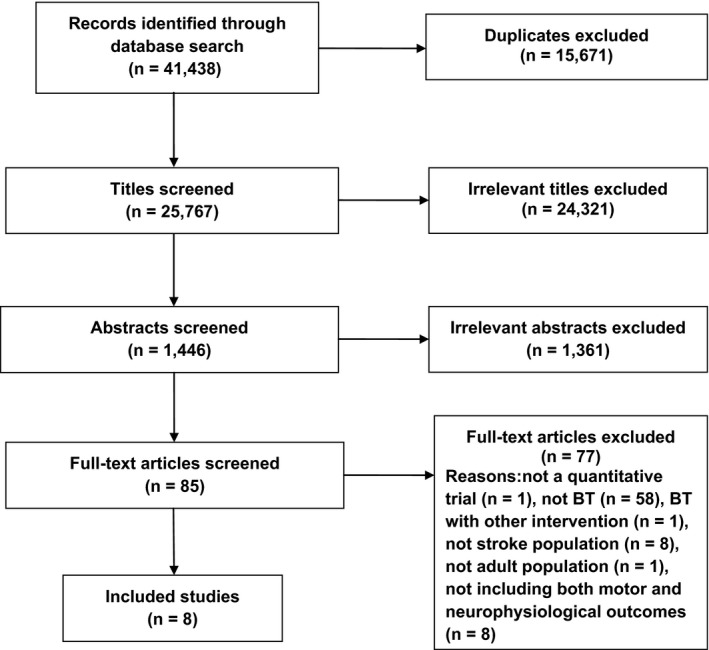
PRISMA flowchart showing the identification process of the included papers.

From a total of 41,438 titles, eight studies comprising 164 participants were identified for inclusion in this systematic review. The PRISMA flow chart is presented in Figure 1. Characteristics of the included studies are described in Table 1, while the TMS and fMRI methodologies are described in Tables 2 and 3, respectively.

**Table 1:** Characteristics of included studies

Study (design; comparison, hypothesis)	Participants (number; age (years); gender (M/F); hand dominance (L/R); time since stroke (years); side of affected hemisphere (L/R); type of stroke; site of stroke, Fugl‐Meyer arm score at baseline).	Intervention (mode of BT; task; amount of practice; organization of practice)	Outcome (time points; outcome measures)
Lewis and Byblow 2004 ^TMS^ Design: NR Comparison: Unilateral versus Bilateral Hypothesis: NR	Stroke group (*n* = 6) Age, median (range): 55.5 (42–84) Gender: 4/2 Hand dominance: NR Time since stroke, median (range): 0.8 (0.1–3.9) Side of stroke: 1/5 Type of stroke: intracerebral hemorrhage (*n* = 1), NR (*n* = 5) Site of stroke: basal ganglia (*n* = 1), ACA (*n* = 1), parietal lobe (*n* = 1), R hemisphere (*n* = 1), internal capsule (*n* = 1), lenticular nucleus (*n* = 1) FM, median (range) – estimated from graphical data: 19 (3–26)	Mode of BT: In‐phase Task: unilateral training of 3 specific tasks using the paretic UL, switching to bilateral training of the same tasks at random start between days 7–13. Selected based on individual functional ability, the 3 tasks were practiced from the following: block placement, peg targeting, peg inversion, cup inversion, rapid transfer and simulated drinking. The same 3 tasks were practiced throughout the study Amount of practice: 11x/task, 5x/week for a total of 20 training days over 4 weeks Organization of practice: NR	Assessment time: 1st: 1 day before intervention, 2nd: after unilateral training (days 7–13) and 3rd: after bilateral training (day 20) Outcome Measures: Motor behavior: FM (UL): hand and forearm components only Rating of task performance using 6–7 criteria: speed, smoothness, path directness, intralimb synchrony, graspquality, joint angles at target and task accuracy (for block placement and cup inversion tasks only) TMS: Map Area for FDI Map Area for ECR Rest threshold (%) Total number of iMEPS from both muscles
Lewis and Perreault 2007 ^TMS^ Design: NR Comparisons: Motor behavior conditions: Unilateral (L, R) Bilateral (in‐phase, anti‐phase) TMS conditions at stimulus intensities 80%, 100%, 120% and 140% resting threshold: Muscle activation conditions: Both arms at rest Arm contralateral to stimulation activated Arm ipsilateral to stimulation activated Both arms activated Hypotheses: interlimb coupling is mediated by: (1) a structure in the L hemisphere; (2) activation of ipsilesional motor pathways	Stroke group (*n* = 15): Age, mean (SD): 58 (14) Gender: 11/4 Hand dominance: 0/15 Time since stroke, mean (SD): 8 (7) Side of stroke: 8/7 Type of stroke: NR Site of stroke: parietal and occipital lobes (*n* = 1), frontal and parietal lobes (*n* = 5), middle internal capsule (*n* = 1), frontal and temporal lobes (*n* = 1), frontal lobe, internal capsule and corona radiata (*n* = 1), posterior internal capsule (*n* = 1), internal capsule (*n* = 2), frontal, parietal and temporal lobes (*n* = 1) and NR (*n* = 2) FM, mean (SD): 42/66 (12)	Motor behavior session: Mode of BT: in‐phase, anti‐phase Task: continuous pronation‐supination of forearm to maximum ROM in splint. Movements were paced by a metronome set at 0.2 Hz below the frequency movements became unstable Amount of practice: three trials of 35s duration for each motor behavior condition Organization of practice: random order TMS session: Task: isometric contraction of muscle biceps brachii to 20 ± 2% of MVC in each of four muscle activation conditions and stimulus intensities (see L‐hand column). Amount of practice: 10 TMS responses for each combination of muscle activation condition and stimulus intensity Organization of practice: random order	Assessment time: 2 separate sessions for motor behavior and TMS Outcome Measures: Motor behavior: Movement amplitude peaks CV of movement amplitude Uniformity of relative phase between the two arms TMS: Number of MEP active positions MEP latency Maximum peak‐to‐peak MEP amplitude
Luft et al. 2004 ^fMRI^ Design: Sub study of an RCT comprising participants who underwent MRI Comparison: BATRAC versus DMTE Hypothesis: BATRAC may be associated with reorganization of brain regions involved in motor control	BATRAC (*n* = 9): Age, mean (SD): 63.3 (15.3) Gender: 7/2 Hand dominance: NR Time since stroke, median (IQR): 6.3 (3.2–7.0) Side of stroke: 3/6 Type of stroke: ischemic (*n* = 9) Site of stroke: cortical (*n* = 6), subcortical (*n* = 2), brainstem (*n* = 1) FM, mean (SE): 29.6/66 (3.5) DMTE (*n* = 12): Age, mean (SD): 59.6 (10.5) Gender: 5/7 Hand dominance: NR Time since stroke, median (IQR): 3.8 (1.9–5.5) Side of stroke: 4/8 Type of stroke: ischemic (*n* = 12) Site of stroke: cortical (*n* = 6), subcortical (*n* = 4), brainstem (*n* = 2) FM, mean (SE): 28.3/66 (3.1)	BATRAC: Mode of BT: in‐phase or anti‐phase Task: bilateral pushing/pulling 2 T‐bar handles in transverse plane upon auditory cues at individually determined rates between 0.67 and 0.97 Hz Amount of practice: 1 h (20‐min practice, 40 min rest)/day, 3x/week for 6 weeks Organization of practice: 4 5‐min movement periods interspersed with 10‐min rest periods DMTE: Task: exercises based on neurodevelopmental principles, including thoracic spine mobilization, scapular mobilization, weight bearing with paretic arm and opening a closed fistAmount of practice: same as for BATRAC	Assessment time: Within 2 weeks before and after intervention. Outcome measures: Motor behavior: FM (UL) WMFT (time, weight) dynamometry (elbow, shoulder peak force) UMAQS fMRI during unilateral elbow flexion–extension upon auditory cues: Number of activated voxels Difference maps identifying activated voxels after intervention
Stinear and Byblow 2004 ^TMS^ Design: NR Comparison: In‐phase Anti‐phase Hypothesis: NR	In‐phase group (*n* = 5): Age, mean (range): 59.6 (55–64) Gender: 3/2 Hand dominance: 0/5 Time since stroke, median (range): 1.0 (0.2–7.0) Side of stroke: 3/2 Type of stroke: ischemic (*n* = 4), hemorrhagic (*n* = 1) Site of stroke: cortical (*n* = 2), capsular (*n* = 2), basal ganglia (*n* = 1) FM at T1, median (range) – estimated from graphical data: 18/30 (3–24) Anti‐phase group (*n* = 4): Age, mean (range): 65.3 (48–84) Gender: 4/0 Hand dominance: 0/4 Time since stroke, median (range): 0.5 (0.2–1.3) Side of stroke: 3/1 Type of stroke: ischemic (*n* = 4) Site of stroke: cortical (*n* = 3), capsular (*n* = 1) FM at T1, median (range) – estimated from graphical data: 5/30 (3–22)	In‐phase (APBT) Mode of BT: in‐phase Task: rhythmical active flexion–extension of the nonparetic wrist driving passive flexion–extension of the paretic wrist, using a purpose‐built manipulandum that mechanically couples both wrists (self‐paced at about ~1.2 Hz) Amount of practice: 1 h/day (divided into sessions of minimum 10 min each) for 4 weeks Organization of practice: N/A Anti‐phase (APBT) Mode of BT: anti‐phase (phase lag about 200 ms–60°) Task: as for in‐phase APBT Amount of practice: as for in‐phase APBT Organization of practice: N/A	Assessment time: 2 baselines (at least 7 days apart) and after 4‐week intervention. FM was reassessed 1 month after intervention in five participants with >10% improvement in FM score over 4‐week intervention. Outcome Measures: Motor behavior: FM (UL): wrist, hand and coordination components only Isometric force (grip strength, wrist extensor strength, wrist flexor strength) TMS: Map CoG Mean muscle map volume
Stinear et al. 2014 ^TMS^ Design: RCT Comparison: Priming with APBP versus priming with TENS Hypothesis: APBP before UL therapy accelerates recovery of function, with a greater proportion of PRIMED participants reaching maximum recovery by 12 weeks	Priming with APBP group (*n* = 29) Age, median (range): 68 (33–97) Gender: 11/18 Hand dominance: NR Time since stroke: within 26 days of stroke Side of stroke: NR Type of stroke: ischemic (*n* = 29) Site of stroke: cortical (*n* = 4), subcortical (*n* = 18), basal ganglia (*n* = 10), brain stem (*n* = 7) FM, median (range): 44 (2–65) Priming with TENS group (*n* = 28) Age, median (range): 71 (31–90) Gender: 15/13 Hand dominance: NR Time since stroke: within 26 days of stroke Side of stroke: NR Type of stroke: ischemic (*n* = 28) Site of stroke: cortical (*n* = 2), subcortical (*n* = 24), basal ganglia (*n* = 15), brain stem (*n* = 2) FM, median (range): 43 (3–64)	Priming with APBP before UL therapy Mode of BT: in‐phase Task: rhythmical active flexion–extension of the nonparetic wrist driving passive flexion–extension of the paretic wrist, using a device that mechanically couples both wrists (self‐paced) Amount of practice: 15 mins/weekday (target of 500–1500 movement cycles, depending on ability) for 4 weeks Organization of practice: block, constant Priming with TENS before UL therapy Task: TENS of volar aspect of paretic forearm Amount of practice: 15 sec/min for 15 min/weekday (including 2s ramp‐up, 2s ramp‐down) for 4 weeks Organization of practice: N/A	Assessment time: Baseline (within 26 days of stroke), after 4‐week intervention, 12 and 26 weeks after stroke. Modified Rankin Scale and SIS were only assessed 26 weeks after stroke. Outcome Measures: Motor behavior: FM (UL) ARAT NIHSS Modified Rankin Scale SIS TMS: S‐R slope Percentage of trials that produced iSPs Depth of iSPs
Summers et al. 2007 ^TMS^ Design: RCT Comparison: Unilateral Bilateral Hypothesis: NR	Bilateral (*n* = 6): Age, mean (SD): 63 (16) Gender: 4/2 Hand dominance: NR Time since stroke, mean (range): 6.3 (1.0–16.2) Side of stroke: 2/4 Type of stroke: ischemic (*n* = 2), NR (*n* = 4) Site of stroke: MCA (*n* = 2), cortical lesion (M1) (*n* = 1),internal capsule (*n* = 1), R hemisphere (*n* = 1), L hemisphere (*n* = 1). MAS, mean (SD): 3.99 (2.30) Unilateral (*n* = 6): Age, mean (SD): 60 (14) Gender: 3/3 Hand dominance: NR Time since stroke, mean (range): 4.0 (0.9–10.4) Side of stroke: 1/4, bilateral (*n* = 1) Type of stroke: ischemic (*n* = 2), hemorrhagic (*n* = 1), NR (*n* = 3) Site of stroke: lacunar (*n* = 1), cerebellar intracerebral (*n* = 1), frontal/temporal (*n* = 1), bilateral MCA (*n* = 1), MCA (*n* = 1) and R hemisphere (*n* = 1). MAS, mean (SD): 3.78 (2.26)	Bilateral Mode of BT: In‐phase Task: Lifting two dowels, one in each hand, and placing them on targets located on shelf (height adjusted to each individual) Amount of practice: 50 training trials/day for 6 days (excluding two warm‐up trials using both hands, four unilateral trials using nonparetic then paretic hand and four unilateral trials with each hand) Organization of practice: N/A Unilateral Task: as above using only the paretic hand. Amount of practice: 50 training trials/day for 6 days Organization of practice: N/A	Assessment time:Kinematic: Before and after each training session MAS: Baseline (1 day before intervention) and 1 day after 6‐day intervention TMS: 2 baselines (1 week and 1 day before intervention) and 1 day after 6‐day interventionOutcome Measures: Motor behavior: MAS Kinematics of task performance (transport tangential speed (index LED), movement onset, movement offset, movement time, velocity profile, curvature, elbow angle) TMS: Mean map volume of EDC Map CoG
Whitall et al. 2011 ^fMRI^ Design: Sub study of an RCT comprising participants who underwent MRI Comparison: BATRAC DMTE Hypotheses: BATRAC results in larger and more durable UL functional improvement, mediated through remodeling of bihemispheric motor and/or premotor cortical networks, compared with DMTE.	BATRAC (*n* = 17): Age, mean (SD): 61.2 (13.8) Gender: 7/10 Hand dominance: 2/15 Time since stroke, mean (SD): 3.9 (2.7) Side of stroke: 7/10 Type of stroke: NR Site of stroke: cortical (*n* = 9), subcortical (*n* = 7), brainstem (*n* = 1) FM, mean (SD): 32/66 (12.5) DMTE (*n* = 21): Age, mean (SD): 54.8 (13.1) Gender: 11/10 Hand dominance: 8/13 Time since stroke, mean (SD): 3.3 (2.1) Side of stroke: 8/13 Type of stroke: NR Site of stroke: cortical (*n* = 9), subcortical (*n* = 10), brainstem (*n* = 2) FM, mean (SD): 27/66 (11.6)	BATRAC: Mode of BT: alternate in‐phase and anti‐phase Task: bilateral pushing/pulling 2 T‐bar handles in transverse plane upon auditory cues set by metronome at preferred speed Amount of practice: 1 h (20‐min practice, 40 min rest)/day, 3x/week for 6 weeks Organization of practice: 4 5‐min movement periods interspersed with 10‐min rest DMTE: Task: 4 exercises based on neurodevelopmental principles, including thoracic spine mobilization with weight shifting, scapular mobilization, weight bearing with paretic arm (elbow fixed) and opening hand with finger extension. Amount of practice: 1 h (20‐min practice, 40 mins rest)/day, 3x/week for 6 weeks Organization of practice: 4 5‐min movement periods interspersed with 10‐min rest	Assessment time: 2 baselines (6 weeks apart), after 6‐week intervention and 4 months after intervention Outcome Measures: Motor behavior: FM (UL) WMFT (time, weight, grip strength, performance) Dynamometry (isokinetic strength of elbow flexion/extension of both arms, isometric strength of both arms and hands) Range of motion (shoulder flexion/extension/abduction, elbow flexion/extension, wrist flexion/extension and thumb opposition) SIS Verbal assessment of participants' perceptions using 5‐point Likert scale on satisfaction with training and improvement after training fMRI during unilateral elbow flexion–extension upon auditory cues: Number of activated voxels
Wu et al. 2010 ^fMRI^ Design: Sub study of an RCT comprising participants who underwent MRI Comparison: BT dCIT Hypothesis: NR	BT (*n* = 4): Age, median (range): 54.5 (45–57) Gender: 4/0 Hand dominance: NR Time since stroke, median (range): 1.0 (0.8–4.8) Side of stroke: 2/2 Type of stroke: ischemic (*n* = 3), hemorrhagic (*n* = 1) Site of stroke: putamen and corona radiata (*n* = 1), corona radiata (*n* = 1), lacunar and thalamus (*n* = 1), thalamus and corona radiata (*n* = 1) FM median (range): 51/66 (38–53) dCIT (*n* = 2): Age, median (range): 62.5 (57–68) Gender: 1/1 Hand dominance: NR Time since stroke, median (range): 2.1 (0.8–3.3) Side of stroke: 0/2 Type of stroke: hemorrhagic (*n* = 2) Site of stroke: thalamus (*n* = 2) FM median (range): 43.5/66 (39–48)	BT: Mode of BT: in‐phase or anti‐phase Tasks: lifting 2 cups, picking up 2 pegs, reaching forward/upward to move blocks, and grasping/releasing 2 towels Amount of practice: 2 h/day, 5x/week for 3 weeks Organization of practice: NR dCIT: Tasks: intensive training of paretic UL with functional activities and behavioral shaping while restricting nonparetic hand with a mitt. Functional activities included reaching forward/upward to move a cup, picking up coins, picking up a utensil to eat food, and grasping/releasing various blocks Amount of practice: 2 h/day, 5x/week for 3 weeks Organization of practice: NR	Assessment time: Before and after 3‐week intervention Outcome Measures: Motor behavior: FM (UL) ARAT MAL (AOU and QOM) fMRI during unilateral finger flexion–extension, and bilateral elbow flexion–extension: Number of activated voxels Laterality index

ACA, anterior cerebral artery; AOU, amount of use; APBT, active‐passive bilateral therapy; ARAT, action research arm test; BATRAC, bilateral arm training with rhythmic auditory cueing; BT, bilateral training; CoG, map center of gravity; CV, coefficient of variation; dCIT, distributed constraint‐induced therapy; DMTE, dose‐matched therapeutic exercises; ECR, extensor carpi radialis; EDC, extensor digitorum communis; FDI, first dorsal interosseous muscles; fMRI, functional magnetic resonance imaging; FM, Fugl‐Meyer arm score; iMEPs, ipsilateral motor evoked potentials evoked by stimulation over the intact hemisphere; IQR, interquartile range; L/R, left/right; iSPs, ipsilateral silent periods; MAL, motor activity log; MAS, Modified Motor Assessment Scale; MCA, middle cerebral artery; MEPs, motor evoked potentials; M/F, male/female; N/A, not applicable; NIHSS, National Institutes of Health Stroke Scale; NR, not reported; QOM, quality of movement; RCT, randomized controlled trial; ROM, range of motion; SD, standard deviation; SICI, short latency intracortical inhibition within contralesional M1; SIS, Stroke Impact Scale; S‐R, stimulus–response; TCI, transcallosal inhibition (TCI) from ipsilesional M1 to contralesional M1; TENS, transcutaneous electrical nerve stimulation; TMS, transcranial magnetic stimulation; UL, upper limb; UMAQS, University of Maryland Arm Questionnaire for Stroke; UT, unilateral training; WMFT, Wolf Motor Function Test.

**Table 2:** Transcranial magnetic stimulation methodology of included papers

Study	Coil Shape/Diameter/Orientation	Description of Grid	Determination of “Hot Spot”	Determination of Resting Excitability Thresholds (RET)	Hemisphere Stimulated and Target Muscles	Muscle Contraction during Stimulation	EMG	Measure on Excitability	Measure on Active Site Mapping
Lewis and Byblow 2004 ^TMS^	70 mm figure of eight coil Tangential to scalp surface with handle angled 45° from midline and pointing backwards, to induce current flow in posterior to anterior direction	Cotton cap with premarked grid locations Size of grid not reported Maintained congruence between vertex and cap position	Scalp location eliciting MEPs of the largest amplitude in ECR and FDI	RET of two muscles determined as the lowest stimulus intensity that generated responses of >50 *μ*V in at least 4 of a train of 8 stimuli	Lesioned and nonlesioned hemispheres to affected muscles ECR FDI	Nonlesioned hemispheres to affected muscles stimulation: 5–10% max voluntary contraction in both muscles Visual feedback provided to assist maintaining contraction level	Sampled at 4000 Hz Amplified and band‐pass filtered (30–1000 Hz)	Not done – map area only	Stimulation of 21 grid locations surrounding the hot spot Lesioned hemisphere mapped at intensity 120% of highest rest threshold of the two muscles Nonlesioned hemisphere at 150% highest rest threshold of the two muscles
Lewis and Perreault 2007 ^TMS^	70 mm figure of eight coil Tangential to scalp surface with handle angled 45° from midline and pointing backwards, to induce current flow in posterior to anterior direction	Not reported	Optimal location for eliciting a MEP in contralateral BB	RET determined as minimum stimulus intensity at which a response greater than 50 *μ*V elicited in at least 4 of a train of 8 stimuli while BB relaxes. If responses could not be elicited at intensities below 70% maximum stimulator output (MSO) then rest threshold defined as 70% MSO.	Lesioned and nonlesioned hemispheres to affected and nonaffected muscles Biceps brachii	Isometric contraction of BB to 20 ± 2% of max voluntary contraction Visual display provided and stimuli not delivered until right level achieved Four muscle activation conditions: Both arms at rest Arm contralateral to stimulation activated Arm ipsilateral to stimulation activated Both arms activated	Sampled at 5000 Hz High and low pass cut off frequencies of 10 and 1000 Hz	MEP latency ipsilateral pathway from lesioned MEP latency contralesional pathway from lesioned MEP latency ipsilateral pathway from nonlesioned MEP latency contralesional pathway from nonlesioned	Not done
Stinear and Byblow 2004 ^TMS^	70 mm figure of eight coil Tangentially to scalp with handle held posterior and orthogonal to assumed plane of the central sulcus	Tight fitting cotton cap with premarked coordinates in a 1‐cm grid pattern Before each session measured the point of intersection on scalp for interaural and nasion‐inion lines for taping in place.	Coordinate where MEP amplitudes were greater than amplitudes of adjacent coordinates for a given stimulus intensity	Highest stimulus intensity delivered over the hot spot that produced no more than 4 of 8 consecutive MEPs with an amplitude of ~ 50 *μ*V while the FCR was at rest	Lesioned hemisphere to affected muscles and nonlesioned hemispheres to nonaffected muscles ECR FCR	No contraction	Sampled at 4000 Hz Band‐pass filtered at 30 to 1000 KHz	Map CoG Mean muscle map volume	6 MEPs collected from FCR for each coordinate of the grid surrounding a position 4 cm lateral to the vertex
Stinear et al. 2014 ^TMS^	70 mm figure of eight coil Coil oriented to induce posterior‐to‐anterior current flow in each M1	Not reported	Not reported	Minimum stimulus intensity that produced a MEP with amplitude ≥50 *μ*V on at least 4 of 8 consecutive trials. RMT was set to 100% maximum stimulator output for participants who did not meet this criterion.	Lesioned hemisphere to affected muscles and nonlesioned hemispheres to nonaffected muscles ECR	No contraction for S‐R slopes. For iSPs, full voluntary extension of ipsilateral wrist against gravity, with rests provided between stimuli as required.	Sampled at 2000 Hz Amplified 1000 gain, filtered 20 to 1000 Hz	S‐R slope for ipsilesional M1 S‐R slope for contralesional M1	Not done but determined percentage of trials that produced and depth of iSPs
Summers et al. 2007 ^TMS^	70 mm figure of eight coil Perpendicular orientation with respect to the presumed direction of the central sulcus	Grid of 1 cm markings based on latitude/longitude system originating at the vertex	Optimal site for producing MEPs in contralateral EDC	Minimum stimulus intensity required to produce discernible MEPs of at least 50 *μ*V peak‐to‐peak in 6 of 10 trials determined for each hemisphere	Lesioned and nonlesioned hemispheres to affected and nonaffected muscles EDC	No contraction	Sampled at 2000 Hz Filter settings: 10 Hz/1000 Hz	Mean map volume of EDC Map CoG	Stated with the hot spot and expanded to surrounding points on grid until sites were reached which did not elicit any activation of EDC

BB, biceps brachii; CoG, map center of gravity; ECR, extensor carpi radialis; EDC, extensor digitorum communis; EMG, electromyography; FCR, flexor carpi radialis; FDI, first dorsal interosseous muscles; iSPs, ipsilateral silent periods; MEPs, motor evoked potentials; S‐R, stimulus–response.

**Table 3:** The fMRI methodology of included papers

Study	Design	Testing task	Comparisons	Control for attention	Control for mirror movements	Data analysis	Time points
Luft et al. 2004 ^fMRI^	Epoch	Task: Active elbow flexion from 45° ending 60–75° depending on movement ability. Strapped device ensured single plane movement and defined ROM. Cue: Once every 3 sec with beeping	Affected versus nonaffected UL Rest versus movement BATRAC versus DMTE Pre versus Post	Participants kept their eyes closed	Video monitoring and taping with 2 cameras on head and elbow to assess compliance with the requested movement. Screened for mirror movement of biceps and deltoid during isometric contraction of affected UL (i.e., synkinesia index).	Whole brain analysis 9 ROIs selected manually	Within 2 weeks pre and post intervention
Whitall et al. 2011 ^fMRI^	Epoch	Task: Active elbow flexion from 45° ending 60–75° depending on movement ability. Strapped device ensured single plane movement and defined ROM. Cue: Once every 3 sec with beeping	Affected versus nonaffected UL Rest versus movement BATRAC versus DMTE Pre versus Post	Not reported	Video monitoring and taping with 2 cameras on head and elbow to assess compliance with the requested movement.	Whole brain analysis 8 ROIs selected manually	6 weeks after first baseline and after 6 weeks of intervention
Wu et al. 2010 ^fMRI^	Epoch	Active finger flexion/extension on affected and nonaffected UL at 2/3 Hz Active bilateral elbow flexion/extension at 1/3 Hz	Affected versus nonaffected UL Rest versus movement BATRAC versus DMTE Pre versus Post	Not reported	Not done.	Whole brain analysis 4 ROIs	Before and immediately after intervention

BATRAC, bilateral arm training with rhythmic auditory cueing; DMTE, dose‐matched therapeutic exercises; ROIs, regions of interest; ROM, range of motion; UL; upper limb.

### Design

Four of the eight studies were reported as RCTs (Luft et al. 2004; Wu et al. 2010; Whitall et al. 2011; Stinear et al. 2014). Four studies did not report study design (Lewis and Byblow 2004; Stinear and Byblow 2004; Lewis and Perreault 2007; Summers et al. 2007). Of these, one used an interrupted time series (Lewis and Byblow 2004) and two used an RCT design (Stinear and Byblow 2004; Summers et al. 2007). The design for the remaining study was unclear (Lewis and Perreault 2007).

### Participants

A total of 164 participants were involved in the included studies. Their demographic and clinical characteristics can be found in Table 1. A mean of 21 participants were included per study (range 6–57). The reported mean age varied between 52.8 and 65.3 years (range 31–97), 55% were male and 62% were more than 3 months after stroke. Side of stroke was reported in seven studies (Lewis and Byblow 2004; Luft et al. 2004; Stinear and Byblow 2004; Lewis and Perreault 2007; Summers et al. 2007; Wu et al. 2010; Whitall et al. 2011), for 107 participants. Of these, 42 (39%) had a left hemisphere stroke, 64 (60%) had a right hemisphere stroke and 1 (1%) had a bilateral stroke. One study reported that 23 (40%) participants had a stroke in the dominant hemisphere (Stinear et al. 2014). Information relating to the type of stroke could not be synthesized due to varied methods used for classification, while precise lesion sites were rarely reported (Table 1). The participants of seven of the eight studies had different initial levels of UL severity (Lewis and Byblow 2004; Luft et al. 2004; Stinear and Byblow 2004; Lewis and Perreault 2007; Summers et al. 2007; Whitall et al. 2011; Stinear et al. 2014), while those in the remaining study had only mild UL impairment (Wu et al. 2010).

### BT interventions

#### Content

The mode of BT in the included studies varied considerably: three studies involved only the in‐phase mode of BT (Lewis and Byblow 2004; Summers et al. 2007; Stinear et al. 2014), four involved both in‐phase and anti‐phase modes (Luft et al. 2004; Lewis and Perreault 2007; Wu et al. 2010; Whitall et al. 2011) and one study used in‐phase or anti‐phase BT (Stinear and Byblow 2004). Of these five studies, only three specified the sequencing of BT practice modes (Stinear and Byblow 2004; Lewis and Perreault 2007; Whitall et al. 2011).

Three studies involved functional tasks, including reaching and grasping objects (Wu et al. 2010), peg targeting/inversion (Lewis and Byblow 2004) and dowel placement (Summers et al. 2007). The remaining five focused on specific UL movements. One of these involved “free” forearm pronation/supination (Lewis and Perreault 2007), while in the remaining four studies, the ULs were mechanically coupled (Luft et al. 2004; Stinear and Byblow 2004; Whitall et al. 2011; Stinear et al. 2014). Two of these studies used bilateral arm training with rhythmic auditory cueing (BATRAC), during which the participant bilaterally pulled and pushed two T‐bar handles upon auditory cues (Luft et al. 2004; Whitall et al. 2011). During Active‐Passive Bilateral Therapy (APBT, Stinear and Byblow 2004) and Active‐Passive Bilateral Priming (APBP, Stinear et al. 2014), the participant used a purpose‐built manipulandum to passively flex/extend their paretic wrist through active, rhythmical flexion/extension of their nonparetic wrist. In contrast to APBT (Stinear and Byblow 2004), which was a stand‐alone intervention, APBP (Stinear et al. 2014) was utilized as a priming technique before UL physiotherapy.

The interventions of three of the eight studies involved more than one UL movement or task (Lewis and Byblow 2004; Lewis and Perreault 2007; Wu et al. 2010), whereas the other five studies involved only a single movement or task throughout the intervention period (Luft et al. 2004; Stinear and Byblow 2004; Summers et al. 2007; Whitall et al. 2011; Stinear et al. 2014).

#### Dose

The dose of BT varied considerably across the included studies. Aside from the single session study (Lewis and Perreault 2007), the overall duration of the intervention periods in the remaining seven intervention studies ranged from 6 days (Summers et al. 2007) to 6 weeks (Luft et al. 2004; Whitall et al. 2011). The frequency of intervention ranged from 3 days/week (Luft et al. 2004; Whitall et al. 2011) to 7 days/week (Stinear and Byblow 2004). The amount of practice per training session ranged from 33 (Lewis and Byblow 2004) to 50 repetitions/day (Summers et al. 2007), while a target of 500–1500 repetitions was set in the APBP study (Stinear et al. 2014). In other studies, active practice time for the affected UL ranged from 1 h (including 20 min of actual practice) (Luft et al. 2004; Whitall et al. 2011) to 2 h a day (Wu et al. 2010). Lewis and Perreault (2007) quantified the amount of practice for the motor session by time (i.e., 105 sec).

### Comparison interventions

There was considerable variation between the comparison conditions. One study (Lewis and Perreault 2007) compared different modes of unilateral and bilateral UL movements in a single session. Six studies compared the effects of BT with another UL intervention: two compared BT with dose‐matched therapeutic exercises (DMTE) (Luft et al. 2004; Whitall et al. 2011), another two compared BT to unilateral training (Lewis and Byblow 2004; Summers et al. 2007) and one study compared BT to distributed constraint‐induced therapy (dCIT) (Wu et al. 2010). The remaining UL intervention study compared in‐phase with anti‐phase BT (Stinear and Byblow 2004). Only one study used APBP as a priming adjuvant and compared this with transcutaneous electrical nerve stimulation (TENS) (Stinear et al. 2014).

### UL outcome measures and time points

The included studies used different measures at different time points. The Fugl‐Meyer Assessment (FM) was the most common UL outcome measure, used in six of the seven intervention studies (Lewis and Byblow 2004; Luft et al. 2004; Stinear and Byblow 2004; Wu et al. 2010; Whitall et al. 2011; Stinear et al. 2014). However, its use was not standardized; only three of these studies used the entire FM UL assessment (Luft et al. 2004; Wu et al. 2010; Whitall et al. 2011). For the remaining two studies, one (Lewis and Byblow 2004) included only the hand and forearm subsection, while the other (Stinear and Byblow 2004) used the wrist, hand and coordination subsections. In one study, it was unclear which part of the FM UL section had been used (Stinear et al. 2014). Two studies used motion analysis to measure UL kinematics (Lewis and Perreault 2007; Summers et al. 2007).

Only two of the intervention studies included a follow‐up assessment: at 12 and 26 weeks after stroke (Stinear et al. 2014), and 4 months after end of intervention (Whitall et al. 2011). One study included a follow‐up assessment for only those participants (*n* = 5 of 9) who had more than 10% improvement in FM score (Stinear and Byblow 2004).

### Neurophysiological measures

Three studies used fMRI (Luft et al. 2004; Wu et al. 2010; Whitall et al. 2011) and five used TMS (Lewis and Byblow 2004; Stinear and Byblow 2004; Lewis and Perreault 2007; Summers et al. 2007; Stinear et al. 2014). The TMS studies utilized a wide range of variables to assess cortical excitability. Variables used for fMRI and TMS are described in Table 1.

### Methodological quality

The methodological quality of the studies is detailed in Table 4. The global rating for methodological quality was “strong” for two studies (Luft et al. 2004; Stinear et al. 2014), “moderate” for two studies (Lewis and Byblow 2004; Whitall et al. 2011) and “weak” for the remaining four (Stinear and Byblow 2004; Lewis and Perreault 2007; Summers et al. 2007; Wu et al. 2010). None of the eight studies rated “strong” for selection bias and blinding. Six studies rated “strong” for study design (Luft et al. 2004; Stinear and Byblow 2004; Summers et al. 2007; Wu et al. 2010; Whitall et al. 2011; Stinear et al. 2014). Three studies rated “strong” for control of confounders (Luft et al. 2004; Whitall et al. 2011; Stinear et al. 2014), seven rated “strong” for data collection methods (Lewis and Byblow 2004; Luft et al. 2004; Stinear and Byblow 2004; Summers et al. 2007; Wu et al. 2010; Whitall et al. 2011; Stinear et al. 2014), while only one study rated “strong” for withdrawals and dropouts (Luft et al. 2004).

**Table 4:** Methodological quality assessment using the Effective Public Health Practice Project (EPHPP) Tool

Study	Selection bias	Study design	Confounders	Blinding	Data collection methods	Withdrawals and dropouts	Global rating
Lewis and Byblow 2004 ^TMS^	Moderate	Moderate	N/A^a^	Moderate	Strong	Weak	Moderate
Lewis and Perreault 2007 ^TMS^	Weak	Weak	N/A^a^	Moderate	Weak	Weak	Weak
Luft et al. 2004 ^fMRI^	Moderate	Strong	Strong	Moderate	Strong	Strong	Strong
Stinear and Byblow 2004 ^TMS^	Moderate	Strong	Weak	Moderate	Strong	Weak	Weak
Stinear et al. 2014 ^TMS^ Summers et al. 2007 ^TMS^	Moderate Moderate	Strong Strong	Strong Weak	Moderate Moderate	Strong Strong	Moderate Weak	Strong Weak
Whitall et al. 2011 ^fMRI^	Moderate	Strong	Strong	Moderate	Strong	Weak	Moderate
Wu et al. 2010 ^fMRI^	Weak	Strong	Weak	Moderate	Strong	Weak	Weak

a Domain could not be assessed as study was not a between‐group design.

### Neural correlates of bilateral training

This section describes the effects of BT on UL motor outcomes, neural function and the associations between these two types of variables. As the results could not be pooled for reasons explained earlier, only a narrative synthesis will be presented. The effects of BT in the included studies are described in Table 5.

**Table 5:** Effects of bilateral upper limb task training in included studies

Study	Motor outcomes	Neurophysiological outcomes	Association between motor and neurophysiological outcomes
Lewis and Byblow 2004 ^TMS^	For FM scores, no significant difference across the 3 assessment times (*P* = 0.05). For rating of task performance, significant improvement within 2 days of switching to BT were found in simulated drinking (*n* = 2), rapid transfer (*n* = 1), peg targeting (*n* = 1) (*P* < 0.05) only. Significant reduction in progression was found in block placement task (*n* = 1) with the commencement of BT (*P* < 0.05).	Contralateral motor pathway (lesioned hemisphere to paretic UL): MEPs could only be elicited in 3 participants. Complete data for only 1 of the 3 participants who could elicit MEPs during all sessions: This participant had a decrease in map area of ECR from assessment 1 to 2 (i.e., before‐after UT) and a small increase in the same map area from assessment 2 to 3 (i.e., before‐after BT). The map area of FDI was relatively consistent across all 3 assessments. Ipsilateral motor pathway (contralesional hemisphere to paretic UL): MEPs could be elicited in 5 of the 6 participants. No significant difference in the total number of iMEPS from both muscles across the 3 assessment times (*P* = 0.8).	NR
Lewis and Perreault 2007 ^TMS^	Effect of task conditions: For the paretic arm, post hoc analysis found significant larger CV of movement amplitude in AP compared to UT (*P* = 0.004) only. Significantly higher uniformity of relative phase values in IP compared to AP (*P* = 0.001).	Complete data for only 13 of the 15 participants due to contraindications to TMS Contralateral MEP amplitude: In the paretic arm, no significant difference in MEP amplitude between activation of arm contralateral to stimulation and activation of bilateral arms (*F*_1,12 _= 2, *P* = 0.2). Ipsilateral MEPs: In the paretic arm, no significant difference in number of iMEPs activated between activation of arm ipsilateral to stimulation and activation of bilateral arms (*P* = 0.2).	None of the correlations were significant (*P* > 0.05).
Luft et al. 2004 ^fMRI^	No significant difference in change in any motor outcome between the BATRAC and DMTE groups (*P* > 0.05).	Activation within specific areas: Significant increase in number of activated voxels in the ipsilesional cerebellum (Talairach coordinates 29/‐77/‐29 (posterior lobe of cerebellum)) (mean (SE) = 2611.4 (691.4), *P* < 0.001)^a^ and contralesional medialprecentral gyrus (Talairach coordinates 12/14/61 (BA 6), 13/‐13/61 (BA 6) (mean (SE) =1572.5 (683.2), *P* = 0.02)^a^ for paretic arm movement in the BATRAC group compared to the DMTE group. Activation by hemisphere: Significant increase in number of activated voxels in the contralesional hemisphere for paretic arm movement in the BATRAC group compared to the DMTE group (mean (SE) = 1253.8 (562.0), *P* = 0.03).	NR Subanalyses using data from 6 of 9 BATRAC participants who showed before‐after differences in fMRI activation found significant increase in FM scores (mean (95% CI) = 4.83 (1.02 to 8.65), *P* = 0.02).
Stinear and Byblow 2004 ^TMS^	Whole Group Significant improvement in FM for all participants in the baseline period (T1 to T2; *P* = 0.04) and in the intervention period (T2 to POST; *P* = 0.02). No significant changes in isometric grip strength, wrist extensor strength and wrist flexor strength. Each Group In‐phase: 3 of 5 participants increased Fugl‐ Meyer score by > 10% Anti‐phase group: 2 of out 4 participants increased Fugl‐ Meyer score by > 10%	Complete data for only 3 of the 9 participants in some comparisons. Whole Group Significant decrease in mean map volume of the unaffected hemisphere between T1 (7.4) and POST (6.1) (*P* = 0.04), but not between T2 (7.0) and POST (6.1) (*P* = 0.07). For all participants with increases in FM score of >10% (*n* = 5), significant decrease in mean map volume of the unaffected hemisphere at POST compared to baseline assessment (5.7) (*P* = 0.030).	Whole Group No significant correlation between changes in map volume and Fugl‐ Meyer scores between T2 and POST.
Stinear et al. 2014 ^TMS^ Summers et al. 2007 ^TMS^	Significantly greater proportion of participants in the priming with APBP group (*n* = 23/29) achieved a plateau of UL recovery at 12 weeks than the priming with TENS group (*n* = 15/28) (intention to treat analysis: *χ* ^2 ^= 4.25, *P* = 0.039; odds ratio 3.32, 95% CI: 1.1–10.7). No significant differences in modified Rankin Scale (*P* > 0.4) and SIS (*P* > 0.2) in priming with APBP group compared to priming with TENS group. Analysis for FM not reported. Significant improvement in MAS in bilateral group compared to unilateral group (*F*_1,10_, *P* = 0.0094). No significant differences in kinematic measures in bilateral group compared to unilateral group (*P* > 0.05).	For the APBP priming group only, ipsilesional S‐R slope increased and was higher at 12 and 26 weeks than at 2 weeks after stroke (12 weeks 0.20 s.d. 0.07 mV/10%MSO, *t*_24 _= 2.34, *P* = 0.028; 26 weeks 0.28 SD 0.08 mV/10% MSO, *t*_24 _= 2.36, *P* = 0.027). For the APBP priming group only, contralesional S‐R slope decreased and was lower at 26 weeks than at 2 weeks after stroke (26 weeks 0.82 SD 0.09 mV/10% MSO, *t*_24 _= 2.82, *P* = 0.010). Analysis for 30 of 57 participants (15 in each group): Percentage of trials that produced iSPs by stimulating ipsilesional M1 (iPS persistence) increased in the ipsilesional hemisphere for the APBP priming group and decreased for the TENS priming group, becoming significantly different by 26 weeks after stroke (APBP: 87% SEM 4%, TENS: 65% SEM 7%, *t*_28 _= 2.78, *P* = 0.011). Depth of iSPs produced by stimulating ipsilesional M1 increased in the ipsilesional hemisphere in the APBP group, and decreased in TENS group, becoming significantly different by 26 weeks after stroke (APBP: 54% SEM 3%, TENS: 44% SEM 3%, *t*_28 _= 2.31, *P* = 0.028). Complete data for only 6 of the 12 participants due to technical difficulty, unusable data and contraindications. Map CoG of EDC significantly more medial (4.51 cm) in the affected than nonaffected (4.17 cm) hemisphere (*P* = 0.0014) in 6/12 participants. No significant differences in any other variables (*P* > 0.05).	NR Significant negative correlation between change in map volume for the contralesional hemisphere only and change in MAS score (rho=‐0.883, p = 0.02) in 6 of 12 participants.
Whitall et al. 2011 ^fMRI^	No UL motor outcome data for fMRI subcohort. Data for entire cohort: Significant improvement in paretic wrist extension in BATRAC group compared to DMTE group (*P* < 0.05).	Data for fMRI subcohort: Activation within specific areas: Significant increase in number of activated voxels in ipsilesional precentral gyrus (*P* = 0.011^a^), contralesional superior frontal gyrus (*P* = 0.012^a^), ipsilesional SMA (*P* = 0.039), ACC (*P* = 0.036) and postcentral gyrus (*P* = 0.023) for paretic arm movement in the BATRAC group compared to the DMTE group.	Within BATRAC participants only, increase in number of activated voxels in the contralesional superior frontal gyrus (*r* = −0.62, *P* = 0.010^a^), bilateral ACC (ipsilesional: *r* = −0.76, *P* = 0.001^a^, contralesional: *r* = −0.68, *P* = 0.004^a^) and bilateral supramarginal gyrus (ipsilesional: *r* = −0.64, *P* = 0.007, contralesional: *r* = −0.69, *P* = 0.003^a^) correlated with shorter time taken for the paretic arm to complete the WMFT. Within DMTE participants only, increase in number of activated voxels in the ipsilesional superior frontal gyrus (*r* = −0.12, *P* = 0.045), contralesional supramarginal gyrus (*r* = −0.45, *P* = 0.044) and bilateral postcentral gyrus (ipsilesional: *r* = −0.55, *P* = 0.012, contralesional: *r* = −0.51, *P* = 0.022) correlated with shorter time taken for the paretic arm to complete the WMFT.
Wu et al. 2010 ^fMRI^	For BT group (*n* = 4): Improvement in FM, median (range) = 6 (4–10) Improvement in ARAT, median (range) = 4 (1–11) Improvement in MAL‐AOU, median (range) = 0.35 (−0.01 to 1.38) Improvement in MAL‐QOM, median (range) = 0.24 (−0.04 to 1.44) For dCIT group (*n* = 2): Improvement in FM, median (range) = 8 (6–10) Improvement in ARAT, median (range) = 9 (1–17) Improvement in MAL‐AOU, median (range) = 0.96 (0.89–1.02) Improvement in MAL‐QOM, median (range) = 1.34 (1.03–1.65)	For BT group during paretic hand movement in total ROIs (primary sensorimotor cortex, premotor cortex, SMA and cerebellum) (*n* = 4): Increase in number of activated voxels in: ipsilesional hemisphere, mean: 187.5, in contralesional hemisphere, mean: 62.3 Increase in laterality index, mean: 0.12 For dCIT group during paretic hand movement in total ROIs (primary sensorimotor cortex, premotor cortex, SMA and cerebellum) (*n* = 2): Increase in number of activated voxels in ipsilesional hemisphere, mean: 226.4, in contralesional hemisphere, mean: 126.0 Increase in laterality index, mean: −0.04	NR

ACC, anterior cingulate cortex; AOU, amount of use; AP, anti‐phase; APBT, Active‐Passive Bilateral Therapy; ARAT, Action Research Arm Test; BATRAC, bilateral arm training with rhythmic auditory cueing; BT, bilateral group; CI, confidence interval; CoG, map center of gravity; CV, coefficient of variation; dCIT, distributed constraint‐induced therapy; DMTE, dose‐matched therapeutic exercises; ECR, extensor carpi radialis; EDC, extensor digitorum communis; FDI, first dorsal interosseous muscles; FM, Fugl‐Meyer arm score; fMRI, functional magnetic resonance imaging; iMEPs, ipsilateral motor evoked potentials evoked by stimulation over the intact hemisphere; IP, in‐phase; iSPs, ipsilateral silent periods; MAL, Motor Activity Log; MAS, Modified Motor Assessment Scale; MEPs, motor evoked potentials; NR, not reported; QOM, quality of movement; ROIs, regions of interest; SD, standard deviation; SE, standard error; SICI, short latency intracortical inhibition within contralesional M1; SMA; supplementary motor area; S‐R, stimulus–response slope; TCI, transcallosal inhibition (TCI) from ipsilesional M1 to contralesional M1;TMS, transcranial magnetic stimulation; WMFT, Wolf Motor Function Test.

Magnitude data provided where documented in study.

^1^Data are mean (standard error) except otherwise noted.

a Remained significant after adjustment for multiple correction.

#### BT including either in‐phase or anti‐phase

This cluster of studies included those featuring practice of functional tasks (Lewis and Byblow 2004; Summers et al. 2007) or UL joint movements (Stinear and Byblow 2004; Lewis and Perreault 2007; Stinear et al. 2014).

One study, in which participants commenced with unilateral training (UT) of three functional tasks, followed by bilateral training (BT) of the same tasks, found mixed results (Lewis and Byblow 2004). There were no significant differences in FM scores between the unilateral and bilateral phases (*P* = 0.05). However, comparing video ratings of performance of functional tasks, within‐subject improvements were reported following switching from UT to BT in a minority of participants, while one participant's performance had deteriorated. TMS was used to map the neurophysiological responses to the interventions, but data were complete for one of the six participants only (Table 5).

An RCT comparing UT with BT of a dowel placement task (Summers et al. 2007) found a significant improvement in the Modified Motor Assessment Scale—but not in any other measures—in the BT compared to the UT group (*P* = 0.0094). TMS data were complete for only three of six participants in each group and differences between the two groups were not analyzed. In terms of the association between changes in UL motor outcomes and neurophysiological measures, a significant negative correlation was reported between change in map volume for the contralesional hemisphere and change in MAS score (rho = −0.883, *P* = 0.02)—but not for the ipsilesional hemisphere. This finding related to all six participants, from both UT and BT groups, for whom TMS data were available.

Two other studies comprised APBT (Stinear and Byblow 2004) and APBP (Stinear et al. 2014). Findings from these two studies do not permit synthesis, as one study utilized APBT as a stand‐alone intervention (Stinear and Byblow 2004), whereas the other used APBP as a priming technique (Stinear et al. 2014). In the randomized APBT trial comparing in‐phase with anti‐phase bilateral wrist flexion/extension (Stinear and Byblow 2004), between‐group differences were not presented for UL motor or TMS data. The average FM score of participants in both groups improved over the intervention period (*P* = 0.02) as well as over the preceding baseline period (*P* = 0.04). Using TMS, the authors found no significant difference in mean map volume over the intervention period (*P* = 0.07). The correlation between change in FM score and change in map volume was not significant, however, correlations were not reported for each group separately. The RCT comparing APBP with a control group given Transcutaneous Electrical Nerve Stimulation (Stinear et al. 2014) reported a significantly greater number of participants in the APBP group attaining a plateau of UL recovery at 12 weeks than in the control group (*P* = 0.039). Those in the APBP group were also three times more likely to achieve this plateau over the same period than those in the control group (OR 3.32, 95% CI 1.1–10.7), however, actual UL outcome data were not presented. No other significant differences were reported. Compared to the control group, where no significant differences were found following the intervention, intrahemispheric cortico‐motor excitability in the APBP group was reported to be significantly increased in the ipsilesional hemisphere (*P* < 0.028) and significantly decreased in the contralesional hemisphere (*P* = 0.010). Interhemipsheric inhibition in the ipsilesional hemisphere showed an increase in the APBP group and a decrease in the control group (*P* < 0.028) after the intervention. However, only 30 of 57 participants (52.6%) were able to maintain paretic wrist extension against gravity, a requirement for this test. The authors did not report any statistical associations between UL motor and neurophysiological outcomes of BT.

The single‐session study comparing different modes of unilateral and bilateral UL forearm pronation and supination found a significantly larger variation in movement amplitude in the bilateral antiphase condition compared to the unilateral condition (*P* = 0.004) and significantly higher uniformity of relative phase values in bilateral in‐phase compared to antiphase tasks (*P* = 0.001) (Lewis and Perreault 2007). The correlations between FM score and measures of cortical excitability were all nonsignificant.

#### BT including a mix of in‐phase and anti‐phase

This cluster of studies included those featuring practice of functional tasks (Wu et al. 2010) or UL joint movements (Luft et al. 2004; Whitall et al. 2011).

In an RCT comparing BT, involving a range of functional tasks, with distributed Constraint‐Induced Therapy (dCIT), all UL motor outcomes improved for both the BT (*n* = 4) and the dCIT group (*n* = 2) (Wu et al. 2010). Using fMRI during movement of the affected hand, the authors reported an increase in the number of activated voxels in both cerebral hemispheres for both groups. As the sample size was small, only descriptive data were reported.

Two RCTs comparing BATRAC with dose‐matched therapeutic exercise (DMTE) used a comparable methodology (Luft et al. 2004; Whitall et al. 2011). Both identified a significant increase in activated voxels in the ipsilesional precentral gyrus in the BATRAC compared with the DMTE group, however, some of the other findings did not concur (Table 5). One of these RCTs (Luft et al. 2004) found no significant difference in any of the UL motor outcomes between the two groups. Using fMRI, a significant increase was found in the BATRAC compared to the DMTE group in terms of the number of activated voxels in the ipsilesional cerebellum (*P* < 0.001), ipsilesional medial precentral gyrus (*P* = 0.02), and contralesional medial precentral gyrus (*P* = 0.03) for paretic arm movement. A correlation between UL motor and neurophysiological measures was not reported. Subgroup analysis of six of nine BATRAC participants who showed before‐after differences in brain activation (i.e., recruitment of premotor area and primary motor cortex) found a significant increase in FM scores (*P* = 0.02). The second of these RCTs (Whitall et al. 2011) did report a significant improvement in paretic wrist extension for the BATRAC group compared to the DMTE group (*P* < 0.05). Not all participants underwent fMRI and changes in UL motor outcomes of the sub cohorts of both groups that did undergo fMRI were not available, however. A significant increase was reported in the number of activated voxels in the contralesional superior frontal gyrus (*P* = 0.012) and ipsilesional precentral gyrus (*P* = 0.011) for paretic arm movement. Additionally, a significant negative correlation was reported between changes in time taken for the paretic arm to complete the Wolf Motor Function Test (WMFT) and an increase in number of activated voxels in the contralesional superior frontal gyrus, bilateral anterior cingulate Figure 1 cortex, and bilateral supramarginal gyrus for BATRAC participants (*r* ≤ −0.62, *P* ≤ 0.01). No correlation was found in the DMTE group between these variables.

### Adverse events

Adverse events were not reported in any of the eight included studies.

## Discussion

The key findings of the present systematic review are that the neural correlates of UL motor behavior in response to BT after stroke remain unclear. A quantitative synthesis was not possible due to heterogeneity across the included studies for types of BT interventions, comparator interventions and measures. The narrative synthesis is limited further because: (1) only two studies were rated as “strong” for methodological quality and none were “strong” for selection bias or blinding, and (2) only 164 people participated in the eight included studies. Detailed discussion of these key findings is provided below in sections for neural correlates, methodological quality of the evidence base, strengths and limitations of the present review and implications for research and practice.

### Correlations between arm motor behavior and brain structure/function following BT

Drawing conclusions from this body of evidence was hampered by the heterogeneity which contraindicated meta‐analysis. This was compounded by the lack of data available for analysis from individual studies, either due to technical difficulties with TMS (Lewis and Byblow 2004; Lewis and Perreault 2007; Summers et al. 2007; Stinear et al. 2014), lack of outcome data (Wu et al. 2010), or incomparable data sets (Lewis and Byblow 2004; Stinear and Byblow 2004). Statistical associations between changes in UL motor performance and neural function as a result of UL interventions were reported in three of eight studies (Stinear and Byblow 2004; Summers et al. 2007; Whitall et al. 2011). However, only two of those (Stinear and Byblow 2004; Whitall et al. 2011) reported an association particular to BT: one study (Stinear and Byblow 2004) found no significant correlation between FM score and change in map volume, but this included data from both the in‐phase and the anti‐phase BT groups. The other study found a significant correlation between the speed at which the Wolf Motor Function Test was completed and an increase in activation of the contralesional superior frontal gyrus following BATRAC but not DMTE (Whitall et al. 2011).

It is clear from this review that, in order to elucidate the neural processes associated with responses to BT in people with arm impairment due to stroke, several methodological issues (especially data collection methods and reporting) need to be addressed in future studies. These will be discussed in subsequent sections.

It remains unclear whether there are any differences in neural activation between in‐phase BT or anti‐phase BT; the only study in this review to have systematically compared unilateral and bilateral in‐phase and anti‐phase modes (Lewis and Perreault 2007) did not examine brain activity patterns associated with the same arm movements. Evidence was found for more variability in affected arm motor performance during bilateral anti‐phase compared to unilateral arm movement, suggesting differences in neuro‐muscular control between the two modes. Findings were also indicative of stronger coupling between the two arms in the bilateral in‐phase compared to the anti‐phase condition. These findings are not surprising, as healthy individuals perform bilaterally identical (i.e., in‐phase) and unilateral tasks with ease (Franz et al. 1991; Fontaine and Lee 1997; Swinnen 2002). However, bilaterally different (e.g., anti‐phase) movements have demonstrated weaker temporal and spatial coupling compared to bilaterally identical movements (Kelso et al. 1979; Serrien and Swinnen 1999; Swinnen 2002). The rationale for using bilaterally different movements in interventions aimed at improving UL function might therefore be questioned. Another comparison requiring further investigation is auditory‐cued versus nonauditory‐cued BT, as neither of the BATRAC studies (Luft et al. 2004; Whitall et al. 2011) differentiated between the effects of BT and those of auditory cueing, which is known to result in an immediate reduction in spatiotemporal variability of unilateral reaching patterns (Thaut et al. 2002).

Comparing these findings with those of other evidence syntheses of BT after stroke (Stewart et al. 2006; Langhorne et al. 2009; Cauraugh et al. 2010; Coupar et al. 2010; Latimer et al. 2010; Sleimen‐Malkoun et al. 2011; Van Delden et al. 2012; Pollock et al. 2014a) is limited, as only those studies reporting measures of both UL motor behavior and neural function were eligible for the present review. Other reviews that included measures of UL motor behavior and neural function did not focus exclusively on BT as an intervention (Kreisel et al. 2006; Buma et al. 2010) but also concluded that—with the exception of reactivation of the lesioned motor areas as the most consistent predictor of recovery (Calautti et al. 2001; Tombari et al. 2004)—there is no single pattern of neuroplasticity during stroke recovery (Kreisel et al. 2006; Buma et al. 2010). Current evidence from this review indicates that the neural correlates of even a specific intervention (i.e., UL BT) are poorly understood and require further investigation. Essentially, the review reported here contributes to a robust indication that there is currently insufficient evidence on which to delineate any specific pattern of UL motor recovery and brain activity in response to BT after stroke.

### Methodological quality of included studies

The assessment of methodological quality of the studies included in this review clearly indicates the need to strengthen the evidence, as ratings were “strong” for only two of eight studies (Luft et al. 2004; Stinear et al. 2014). Reporting of withdrawals and dropouts, as well as adverse events, was poor and often not in accordance with the CONSORT guidelines (Schulz et al. 2010). Blinding was not rated “strong” in any of the studies, but this could reflect an issue with the EPHPP tool itself, where a “strong” score can only be obtained if, in addition to the assessor being blinded, study participants are not aware of the research question. It is usually not possible to ascertain the latter, resulting in a downgrade of the score on this item.

Another potential contributor to the assessment of methodological quality could be that the studies meeting the inclusion criteria for this systematic review are mostly early phase trials or experimental studies rather than definitive clinical trials. However, in deriving evidence from any study it is still important to consider the potential risk of bias in the results.

A large proportion of missing data, as shown in Table 5, threatens the internal validity of these studies and questions the suitability of the methods used in a stroke population. Using TMS in this population appeared to be particularly challenging and methods that support lower attrition rates than the studies included in this review are required to improve the quality of research in this area. Furthermore, the measures selected to assess neurophysiological variables should be shown to be valid and based on a clear rationale.

### Strengths and limitations of the present review

The methodology for study selection, quality assessment, and data extraction for this review, using independent assessors, was systematic and rigorous. The quality assessment tool used in this review has been methodically chosen for (1) its purpose of assessing primary research designs in systematic reviews, (2) applicability for randomized and nonrandomized studies, (3) its form of a checklist with a summary score, (4) its key domains relevant to the research questions, and (5) evidence of careful development including validity and reliability. There are, however, some limitations in its validation process (i.e., inappropriate methods of assessing levels of agreement between EPHPP and a comparison tool) (Thomas et al. 2004).

This review was limited by the inclusion of English language papers only, a possibility remains that some papers may have been missed. Otherwise, the search strategy was comprehensive.

### Implications for research and practice

The key implication for research and practice is that this review did not find consistent evidence of the neural correlates of UL motor response to BT after stroke. In order to clearly determine the therapeutic potential of different modes of BT for patients with different types of brain lesions, these modes need to be examined systematically. The challenge in this area of research is thus threefold: identifying effective BT training modes, matching the optimal mode to patients with specific stroke lesions and UL impairments, and using valid and appropriate measures based on a clear rationale.

This current review indicates that participant characteristics such as type and precise site of stroke need to be reported more consistently and comprehensively in future studies (Kreisel et al. 2006). For example, diffusion tensor imaging (DTI) could be used to describe corticospinal tract integrity. This information would contribute to a better understanding of motor and neural responses to different BT modes (Van Delden et al. 2012). Future studies should also investigate the influence of stroke chronicity on responses to BT, so as to understand the neurophysiological markers of recovery (Ward et al. 2013).

As mentioned earlier, this review found diversity in BT modes between studies. Clearly, these modes provide different types of sensory input, which may have a differential effect on UL recovery, depending on the integrity of the neural pathways involved. Studies are required to systematically compare different modes of BT in participants with known stroke lesions. Since different modes of BT are thought to exploit distinct neural mechanisms (Cauraugh and Summers 2005), future studies should also explore the unknown relationship between different modes of UL movement and brain activity patterns.

The frequency, intensity, duration, proportion of rest and actual practice, and organization of BT practice were all poorly reported and should be clearly detailed in future studies to enable replication (see Table 1). Additionally, the treatment dose in many of the included studies was low compared to recent studies. A recent Cochrane review suggested that intervention sessions of 30–60 min, 5–7 days a week may provide a treatment effect (Pollock et al. 2014b), whereas Birkenmeier et al. (Birkenmeier et al. 2010) found a few hundred repetitions per session beneficial for stroke participants. Four studies were well below this suggested dosage (Lewis and Byblow 2004; Luft et al. 2004; Summers et al. 2007; Whitall et al. 2011), whereas the APBP priming intervention (Stinear et al. 2014) included 500–1500 repetitions—albeit driven by the nonaffected side. Future intervention studies should aim for an adequate dose of practice where possible.

Comparisons should allow the analysis of effects of BT as a single intervention to understand the effects of BT alone. In this review, two studies compared UL motor outcomes and brain activation between BATRAC and DMTE (Luft et al. 2004; Whitall et al. 2011). While considered a mode of BT, the additional element of rhythmic auditory stimulation, which is embedded within BATRAC, could confound results. In terms of other confounders, one of the three fMRI papers did not control for mirror movements (Table 3) (Wu et al. 2010). FMRI methodology should minimize and control for mirror movements as they can confound bilateral activation patterns (Kim et al. 2003).

Another complication in synthesizing the findings from this review were the wide range and variation of neurophysiological measures in the studies included (Tables 2 and 3). For fMRI, this was mainly due to differences in the analysis methods for the regions of interest (ROI). For the TMS studies, eliciting MEPs in stroke participants appeared to be challenging, which limited the amount of usable data. Additionally, a wide range of TMS measures were used which limited comparison between studies. As sample sizes in future studies on this topic are unlikely to be large, standardization of measures across different studies would facilitate data to be pooled for meta‐analyses, enabling clearer conclusions to be drawn.

In future studies it would also be useful to determine correlations between neural and behavioral measures to shed insight on the neural processes underlying BT. Studies should, however, avoid analyses from inadequately powered subgroups of participants.

Before the pattern of neuroplasticity associated with different BT modes is delineated, the potential value of BT as a therapeutic intervention must not be prematurely cast off, however. Therapists should continue to use clinical reasoning when selecting BT, by considering various modes of delivery in conjunction with patients' UL impairments and functional goals.

## Conflicts of Interest

There are no conflicts of interest to declare.

## Supporting information


**Appendix S1.** Search Strategy.
