# Supplementary material for: Correlations between arm motor behavior and brain function following bilateral arm training after stroke: a systematic review
Source: Brain Behav. 2015 Nov 26;5(12):e00411. doi: 10.1002/brb3.411 (PMC4714643; doi:10.1002/brb3.411)
Supplement: Supplementary file 1 — Appendix S1. Search Strategy. [file BRB3-5-e00411-s001.docx]

**Appendix S1. Search Strategy**

*MEDLINE search strategy*

The following combination of controlled (MeSH) and free-text terms relating to the condition ‘Stroke’, intervention ‘Simultaneous bilateral upper limb training’ and body part ‘Upper limb’ were used. This search strategy was modified to suit other databases (i.e. Cochrane Stroke Trials Register, PsycINFO, ProQuest Central, Web of Science, Physiotherapy Evidence database PEDro, OTseeker and REHABDATA).

1. cerebrovascular disorders/ or exp basal ganglia cerebrovascular disease/ or exp brain ischemia/ or exp carotid artery diseases/ or exp cerebrovascular trauma/ or exp intracranial arterial diseases/ or exp intracranial arteriovenous malformations/ or exp "intracranial embolism and thrombosis"/ or exp intracranial hemorrhages/ or stroke/ or exp brain infarction/ or vasospasm, intracranial/ or vertebral artery dissection/

2. (stroke or poststroke or post-stroke or cerebrovasc$ or brain vasc$ or cerebral vasc$ or cva$ or apoplex$ or SAH).tw.

3. ((brain$ or cerebr$ or cerebell$ or intracran$ or intracerebral) adj5 (isch?emi$ or infarct$ or thrombo$ or emboli$ or occlus$)).tw.

4. ((brain$ or cerebr$ or cerebell$ or intracerebral or intracranial or subarachnoid) adj5 (haemorrhage$ or hemorrhage$ or haematoma$ or hematoma$ or bleed$)).tw.

5. hemiplegia/ or exp paresis/

6. (hemipleg$ or hemipar$ or paresis or paretic).tw.

7. 1 or 2 or 3 or 4 or 5 or 6

8. *cerebrovascular disorders/rh or exp *basal ganglia cerebrovascular disease/rh or exp *brain ischemia/rh or exp *carotid artery diseases/rh or exp *cerebrovascular trauma/rh or exp *intracranial arterial diseases/rh or exp *intracranial arteriovenous malformations/rh or exp *"Intracranial Embolism and Thrombosis"/rh or exp *intracranial hemorrhages/rh or *stroke/rh or exp *brain infarction/rh or *vasospasm, intracranial/rh or *vertebral artery dissection/rh

9. *hemiplegia/rh or exp *paresis/rh

10. 8 or 9

11. exp Upper Extremity/

12. (upper adj3 (limb$ or extremity)).tw.

13. (arm or shoulder or elbow or forearm or hand or wrist or finger or fingers).tw.

14. 11 or 12 or 13

15. rehabilitation/ or "recovery of function"/

16. Physical Therapy Modalities/ or Physical Therapy Specialty/

17. exercise movement techniques/ or exercise/ or exercise therapy/

18. range of motion, articular/ or movement/ or motor activity/ or kinesiology, applied/

19. "Task Performance and Analysis"/

20. occupational therapy/ or activities of daily living/

21. motor skills/ or "physical education and training"/

22. (rehabilitation or recovery of function or physiotherap$ or physical therap$ or exercise$ or movement$ or motor activit$ or occupational therap$ or activities of daily living or adl).tw.

23. ((bilateral or bimanual) adj5 (train$ or retrain$ or facilitat$ or function$ or activit$)).tw.

24. ((mirror$ or coupled) adj5 movement$).tw.

25. 15 or 16 or 17 or 18 or 19 or 20 or 21 or 22 or 23 or 24

26. 10 and 14

27. 7 and 14 and 25

28. 26 or 27

29. limit 28 to humans

*EMBASE search strategy*

1. cerebrovascular disease/ or exp basal ganglion hemorrhage/ or exp brain ischemia/ or exp carotid artery disease/ or cerebrovascular accident/ or exp brain infarction/ or exp cerebrovascular accident/ or exp brain ischemia/ or exp cerebral artery disease/ or brain arteriovenous malformations/ or exp thromboembolism/ or exp brain hemorrhage/ or *brain vasospasm/ or artery dissection/

2. (stroke or poststroke or post-stroke or cerebrovasc$ or brain vasc$ or cerebral vasc$ or cva$ or apoplex$ or SAH).tw.

3. ((brain$ or cerebr$ or cerebell$ or intracran$ or intracerebral) adj5 (isch?emi$ or infarct$ or thrombo$ or emboli$ or occlus$)).tw.

4. ((brain$ or cerebr$ or cerebell$ or intracerebral or intracranial or subarachnoid) adj5 (haemorrhage$ or hemorrhage$ or haematoma$ or hematoma$ or bleed$)).tw.

5. hemiplegia/ or exp paresis/

6. (hemipleg$ or hemipar$ or paresis or paretic).tw.

7. 1 or 2 or 3 or 4 or 5 or 6

8. *cerebrovascular disease/rh or exp *basal ganglion hemorrhage/rh or exp *brain ischemia/rh or exp *carotid artery disease/rh or *cerebrovascular accident/rh or exp *brain infarction/rh or exp *cerebrovascular accident/rh or exp *brain ischemia/rh or exp *cerebral artery disease/rh or *brain arteriovenous malformations/rh or exp *thromboembolism/rh or exp *brain hemorrhage/rh or *brain vasospasm/rh or *artery dissection/rh

9. *hemiplegia/rh or exp *paresis/rh

10. 8 or 9

11. exp arm/

12. (upper adj3 (limb$ or extremity)).tw.

13. (arm or shoulder or elbow or forearm or hand or wrist or finger or fingers).tw.

14. 11 or 12 or 13

15. rehabilitation/ or convalescence/

16. physiotherapy/

17. kinesiotherapy/ or exercise/ or kinesiotherapy/

18. "joint characteristics and functions"/ or "movement (physiology)"/ or motor activity/ or kinesiology/

19. task performance/

20. occupational therapy/ or daily life activity/

21. physical education/ or motor performance/

22. (rehabilitation or recovery of function or physiotherap$ or physical therap$ or exercise$ or movement$ or motor activit$ or occupational therap$ or activities of daily living or adl).tw.

23. ((bilateral or bimanual) adj5 (train$ or retrain$ or facilitat$ or function$ or activit$)).tw.

24. ((mirror$ or coupled) adj5 movement$).tw.

25. 15 or 16 or 17 or 18 or 19 or 20 or 21 or 22 or 23 or 24

26. 10 and 14

27. 7 and 14 and 25

28. 26 or 27

29. limit 28 to human

*CINAHL search strategy*

1. (MH "Cerebrovascular Disorders+")

2. (MH "Carotid Artery Diseases+")

3. (MH "Cerebral Aneurysm")

4. (MH "Intracranial Embolism and Thrombosis")

5. (MH "Cerebral Ischemia+")

6. (MH "Cerebral Vasospasm")

7. (MH "Intracranial Hemorrhage+")

8. (MH "Vertebral Artery Dissections")

9. S1 or S2 or S3 or S4 or S5 or S6 or S7 or S8

10. stroke or poststroke or post-stroke or cerebrovasc* or brain vasc* or cerebral vasc* or cva* or apoplex* or SAH

11. brain* or cerebr* or cerebell* or intracran* or intracerebral n5 isc?emi* or infarct* or thrombo* or emboli* or occlus*

12. brain* or cerebr* pr cerebell* or intracerebral or intracranial or subarachnoid n5 haemorrhage* or hemorrhage* or haematoma* or

bleed*

13. (MH "Hemiplegia") OR (MH "Stroke Patients")

14. hemipleg* or hemipar* or paresis or paretic.tw.

15. S9 or S10 or S11 or S12 or S13 or S14

16. mm cerebrovascular disorders+/rh

17. mh carotid artery diseases+/rh

18. mm cerebral aneurysm/rh

19. mm “cerebral embolism and thrombosis”/rh

20. mh cerebral ischemia+/rh

21. mm cerebral vascular accident/rh

22. mm cerebral vasospasm/rh

23. mh intracranial hemorrhage+/rh

24. mm vertebral artery dissections/rh

25. S16 or S17 or S18 or S19 or S20 or S21 or S22 or S23 or S24

26. mm hemiplegia/rh

27. S25 or S26

28. (MH "Upper Extremity+")

29. upper n3 limb* or extremity.tw.

30. arm or shoulder or elbow or forearm or hand or wrist or finger or fingers.tw.

31. S28 or S29 or S30

32. (MH "Rehabilitation")

33. (MH "Activities of Daily Living")

34. (MH "Home Rehabilitation+")

35. (MH "Occupational Therapy+")

36. (MH "Physical Therapy+")

37. S32 or S33 or S34 or S35 or S36

38. (MH "Occupational Therapists")

39. (MH "Occupational Therapy Assistants")

40. (MH "Physical Therapists")

41. S38 or S39 or S40

42. (MH "Exercise+")

43. (MH "Therapeutic Exercise+")

44. (MH "Exercise Intensity")

45. S42 or S43 or S44

46. (MH "Kinesiology")

47. (MH "Applied Kinesiology")

48. (MH "Recovery")

49. S46 or S47 or S48

50. (MH "Movement")

51. (MH "Motor Activity")

52. (MH "Range of Motion")

53. S50 or S51 or S52

54. (MH "Task Performance and Analysis")

55. (MH "Physical Education and Training")

56. (MH "Motor Skills+")

57. S55 or S56

58. rehabilitation or recovery of function or physiotherapy* or physical therap* or exercise* or movement* or motor active* or

occupational therap* or activities of daily living or adl.tw.

59. bilateral or bimanual n5 train* or retrain* or facilitate* or function* or activit*.tw.

60. mirror* or coupled n5 movemen*.tw.

61. 37 or 41 or 45 or 49 or 53 or 54 or 57 or 58 or 59 or 60

62. 27 and 31

63. 15 and 31 and 61

64. 62 or 63

*AMED search strategy*

1. cerebrovascular disorders/ or cerebral hemorrhage/ or cerebral infarction/ or cerebral ischemia/ or cerebrovascular accident/

2. (stroke or poststroke or post-stroke or cerebrovasc* or brain vasc* or cerebral vasc* or cva* or apoplex* or SAH).tw.

3. ((brain* or cerebr* or cerebell* or intracran* or intracerebral) N5 (isch?emi* or infarct* or thrombo* or emboli* or occlus*)).tw.

4. ((brain* or cerebr* or cerebell* or intracerebral or intracranial or subarachnoid) N5 (haemorrhage* or hemorrhage* or haematoma* or hematoma* or bleed*)).tw.

5. hemiplegia/

6. (hemipleg* or hemipar* or paresis or paretic).tw.

7. S1 or S2 or S3 or S4 or S5 or S6

8. exp arm/

9. (upper N3 (limb* or extremity)).tw.

10. (arm or shoulder or elbow or forearm or hand or wrist or finger or fingers).tw.

11. S8 or S9 or S10

12. rehabilitation techniques/ or ”activities of daily living“/ or exp occupational therapy techniques/

13. movement/ or motor activity/ or ”range of motion“/ or ”recovery of function“/

14. occupational therapists/ or physiotherapists/

15. exp physical therapy modalities/
16. physical therapy speciality/ or occupational therapy speciality

17. exercise/ or exercise movement techniques/ or exercise therapy/

18. movement/ or motor activity/ or ”range of motion“/

19. ”task performance and analysis“/ or applied kinesiology/

20. exp physical education/ or exp motor skills/

21. psychomotor performance/

22. (rehabilitation or recovery of function or physiotherap* or physical therap* or exercise* or movement* or motor activit* or occupational therap* or activities of daily living or adl).tw.

23. ((bilateral or bimanual) N5 (train* or retrain* or facilitat* or function* or activit*)).tw.

24. ((mirror* or coupled) N5 movement*).tw.

25. S12 or S13 or S14 or S15 or S16 or S17 or S18 or S19 or S20 or S21 or S22 or S23 or S24

26. S7 and S11 and S25
